# Gangrene of the Leg, the Result of Injury—Fractures of the Femur—Hemorrhoids, Treatment by Ligature

**Published:** 1883-05

**Authors:** John Ashhurst

**Affiliations:** Professor of Clinical Surgery in the University of Pennsylvania


### BUFFALO

### Medical and Surgical Journal.

Vol. XXII. MAY, 1883. No. 10.

### Original Communications.

### Gangrene of the Leg, The Result of Injury—Fractures of the Femur—Hemorrhoids, Treatment by Ligature.

A CLINICAL LECTURE DELIVERED AT THE HOSPITAL OF THE UNIVERSITY OF PENNSYLVANIA, DECEMBER 2, 1882.

By John Ashhurst, Jr., M. D.,
Professor of Clinical Surgery in the University of Pennsylvania.

Reported for the "Buffalo Medical and Surgical Journal,"
By William H. Morrison, M. D.

Gentlemen—This patient was admitted on Wednesday night; he had been injured some two or three weeks previously by a pile of lumber falling upon his left leg. For some time after the accident, the patient was treated at his home and did well. He was treated by irrigation, which is an excellent mode in cases of contused wounds. After a number of days had elapsed, the part began to swell, and the swelling extended high up the thigh, even more than it does at present. Spots began to appear on different parts of the leg, and soon the foot and the lower portion of the leg fell into a condition of moist gangrene. You see there the appearance of the limb. We have not been able to positively diagnose the existence of a fracture.

As you probably know, there are different forms of gangrene. The most important division is into dry and moist gangrene. Considerable confusion sometimes exists in the minds of students as regards these varieties and as regards their causes. The difference between dry and moist gangrene is simply this: In dry gangrene the death of the part takes place slowly. We see this form in old age from structural changes in the vessels, and as a result of thrombosis and embolism, or of injury, causing obstruction of an artery while the veins are unaffected. The blood is carried from the part by the veins, but owing to the obstruction of the artery, fresh blood cannot enter it and the part dies from want of nutriment. On the other hand, when the death of the part occurs suddenly, as from a violent injury or anything causing obstruction to the venous circulation, moist gangrene occurs. There may at the same time, of course, be obstruction of the arteries.

We have made free incisions, as you see, in different parts of the limb, to allow the escape of the fluids and prevent the spread of the gangrene. I think that we have succeeded. This separation of the cuticle is a characteristic of gangrene, and it is a point which you will sometimes find of service in distinguishing between gangrene and the discoloration resulting from a bruise. In gangrene the cuticle moves freely on the tissues beneath.

We can now see on the leg a line between the dead and living part. That is not the line of separation, but of demarcation. The line of demarcation simply shows where the gangrene has stopped; it marks off the living from the dead. The line of separation does not form until a later period. It is a line of granulations, not, as is sometimes said, a line of ulcerations. The dead part is, as it were, pushed off by the restorative process of granulation; just as in the case of bone the sequestrum is pushed away from the living bone by the granulations. When the line of separation forms, that is when we see a line of granulations coming up between the living and dead parts, we shall feel that an operation is justifiable. As a rule, it is not proper to operate in cases of gangrene until this line is well formed. But there are exceptions to this, as to most other rules. In certain forms of gangrene you are not only justified, but required to operate at an early period. In cases of what is termed acute traumatic gangrene, you are required to operate at a comparatively early period. This may occur after compound fractures, or after severe lacerated wounds. The worst case that I ever saw occurred in a lion-tamer, who had been torn by the beast with which he was experimenting. Acute traumatic gangrene set in, causing death in less than 24 hours.

When this patient was brought to the hospital, the history was that the gangrene had come on slowly, and that the swelling, which extended some distance above the thigh, was decreasing. This was favorable. Watching the case, we found that there was no disposition for the gangrene to spread. We were, therefore, justified in waiting. It would not have been proper to operate; in the first place, because we should not have known at what point it would have been safe to amputate in order to remove all of the part that would eventually die; and in the second place, the violence inflicted by the operation would have tended to increase the risk of gangrene, and we should, in all probability, have had sloughing of the flaps. In these cases the rule is to wait until nature points out where the gangrene is to stop.

If, however, we had found that the gangrene was spreading, we should not have had any right to wait. If it had gotten above the knee in 24 hours, it would probably have gone on until it had reached the trunk, and would have been followed by a fatal result; for, when this form of gangrene reaches the trunk, death occurs almost immediately. We had a case of that kind in the hospital a few months ago. A man had received a severe contusion of the arm, accompanied by a fracture of one of the condyles of the humerus; the arm was greatly bruised. He was treated by irrigation and the case did well for some time, but suddenly he began to complain of violent pain in the arm, blebs began to form and gangrenous spots appeared on the limb. I made free incisions, hoping to prevent the spread of the gangrene. Being compelled to leave the city, I requested Dr. H. R. Wharton to see the case in the evening. At that time he found that the gangrene was spreading so rapidly, that he very properly at once amputated the arm, and the patient recovered. If in that case we had waited for the line of demarcation, we should have waited in vain; the patient would have died. In such cases you must watch carefully and be prepared to operate at once if it is necessary.

In this man we have reason to believe that the disease is limited. When the line of separation has formed, we shall probably amputate. His temperature has ranged between 100° in the morning and 102° in the evening. This is not excessively high, considering the amount of local disease.

There are other forms of gangrene, which are of considerable interest. One form, which is met with more frequently in military than in civil practice, is due to the wounding of the principal artery of the limb. High up, for instance, if a ball wounds the femoral artery, and the patient does not die from hemorrhage, there is risk of gangrene. In such cases the rule laid down by Guthrie is, I think, the proper one. If the disease appears in the foot, as long as it remains below the ankle the surgeon should wait, for in many cases the gangrene is limited; but if it spreads and passes above the ankle, experience has shown that it will not be arrested until it reaches a point a short distance below the knee. Under such circumstances Guthrie's rule is to amputate at the point of election, i. e., a short distance below the tubercle of the tibia. By so doing you save time and risk. Of course, if in civil practice you have a case which can be traced to an injury of the artery high up, the same treatment may be adopted; where, however, the gangrene is due to a local injury, the artery is probably bruised and occluded by clots. In these cases, the rule is to wait for the line of separation.

Then we have senile gangrene, occurring in old people as the result of atheroma, or calcification of the arteries. In this form it is better not to operate at all, for the amputation of even so small a part as a toe, is almost certain to be followed by sloughing of the flaps, and, in all probability, death will eventually ensue. It is better to simply use deodorizing applications and leave the case to the powers of nature. It was advised by the late Mr. James, of Exeter, England, that in senile gangrene the surgeon should at once amputate in the upper third of the thigh. It is, however, evident that it is impossible to say before-hand, in any given case, that such an operation will be required; there is a possibility that the gangrene will be spontaneously arrested. Further, by performing such a serious operation in an old person, you add greatly to the risks, for even in young and healthy persons this is a dangerous operation. My advice is not in favor of this operation. If the part separates, leaving an unsatisfactory stump, it may be proper to strip up the soft parts and remove a portion of the bone, but do not perform any regular formal amputation.

For the purpose of deodorizing gangrenous limbs, there is nothing better than the application of charcoal, which, as you know, acts mechanically by absorbing the offensive emanations. In the present case we employ the charcoal poultice. If it is desired, a fermenting poultice may be made by the use of yeast or porter; or the charcoal may be applied in the form of a powder. (This patient's limb was amputated above the knee a week subsequently, and for a time he did well, but ultimately sank; dying from exhaustion ten days after the operation.)

### FRACTURE OF THE SHAFT OF THE THIGH BONE.

This man has a form of injury of which we see a large number of examples in this hospital. He has a fracture of the shaft of the thigh bone. When admitted there was three-fourths of an inch shortening, marked bowing of the bone outwards and crepitus. He has been under treatment for ten days and is doing well.

One of the common symptoms of fracture of the femur is in this man, not as marked as it is sometimes, that is, effusion into the knee-joint. There is a little effusion here; I find that the patella floats, and that I can press it down, but as soon as I remove my finger, it rebounds. This symptom is almost universally present in fracture of the thigh bone. The explanation of this has been given by Gosselin. The blood effused in the neighborhood of the fracture makes its way downward until it reaches the exterior of the synovial membrane, and by irritation of its external surface produces a positive sub-acute synovitis, followed by effusion into the joint. The swelling subsides spontaneously in the course of a week or ten days, and is, as a rule, a matter of no consequence. It is, however, important that you should know that it is a symptom of fracture, for otherwise, it might give rise to the suspicion that the joint had been injured. It has another bearing of some importance as regards the particular mode of dressing. Many surgeons direct that the extension bands be carried to the seat of fracture and then surround the limb with a circular strip above the knee. If a limb is dressed in this way with the knee covered in, and swelling occurs, it causes pain, and it becomes necessary to remove the dressing. It is unnecessary to carry the extending bands above the knee, for it is a mistake to say that the ligaments of the knee can be stretched by any force which we can apply. No force applied to the skin can specially affect the ligaments.

In regard to the amount of weight, I think that you will generally find two, or at the most, three bricks sufficient. A brick is estimated to weigh four pounds and a half. We begin with one brick. This steadies the limb and prevents spasm; afterwards, we add a second brick and perhaps a third. Some surgeons use very heavy weights, as high as twenty and thirty pounds or even more. There is some risk of producing non-union by such powerful extension. I consider shortening an unavoidable result of fracture of the femur in adults. The union of the fragments of a broken bone may be roughly illustrated by a piece of broken sealing wax. If you wish to unite the two pieces, it can only be done at the expense of the wax itself. In a broken bone the fragments unite by the formation of granulation tissue, cartilaginous callus develops and finally bone is formed; so that a little shortening is inevitable in an adult, though it may be very slight in children, there may be an increased length of the limb. This is not due to a separation of the fragments nor to a stretching of the ligaments, but owing to the irritation there is developed, to an excessive degree, the osteogenetic function of the so-called cartilages of conjunction, and there is an increased production of bone. As far as the fracture is concerned, there has been shortening, but this is more than compensated for by the increased production of bone.

In order to keep a fractured bone at rest, it is necessary to fix both the joint above and below. When a joint is affected you fix the bone above and below. In fracture of the thigh, the knee-joint should be fixed, and, if possible, the hip-joint. I think that this can be best accomplished by the use of the long splint of Liston, or that of Desault as modified by Physick, with the foot-piece removed.

We have here a long splint extending from the axilla to just below the foot, and a short splint extending from the groin to the inner malleolus. The splints are wrapped in a splint-cloth, and between them and the limb are placed these bags filled with bran and known as junk-bags. Many explanations have been given as to the exact derivation of this term "junk" bags. One is that it is obtained from the French word "jonc," which means a reed, from the idea that the older surgeons used reeds in the treatment of fractures. Another explanation is that it is a corruption of the term junction or joining bag. Whatever may be the correct explanation, the fact is, from time immemorial they have been called junk-bags. I prefer the use of the long splints and junk-bags to the use of sand bags, where there is a fracture in the shaft of the bone, for I think that they give a better lateral support. There is always a tendency in this injury to an outward bowing, and I have seen this deformity quite marked after the use of the sand bags. In fractures of the neck of the bone, sand bags will answer very well. This tendency to bring outwards may also be in part overcome by keeping the limb in a state of abduction. The splints are kept in position by means of five strips of bandage; one at the ankle, one at the knee, one as high in the crotch as it is possible to get it, one around the pelvis and another at the upper extremity of the long splint.

### HEMORRHOIDS TREATED BY LIGATION.

The history of this patient is, that for a number of years he has suffered from hemorrhages from the bowel. He at one time lost a great deal of blood, but of late, the loss has not been so great. He is, however, very pale and anæmic. On examining the rectum, I find internal piles. He is anxious to be relieved, and as I can detect no visceral disease, nor any contra-indication, I shall operate. I shall use for this purpose the ordinary nævus needle.

Hemorrhoids or piles are divided into internal and external, or into sub-mucous and sub-cutaneous. Sub-mucous or internal piles, are those which are situated above the sphincter; the purely sub-cutaneous or external piles, are those little masses presenting themselves outside of the anus and covered with skin. A large number, probably the majority, of piles present characters which place them midway between these two varieties. They are partly sub-mucous and partly sub-cutaneous. These are the intero-external, or sub-muco-cutaneous piles.

The treatment differs somewhat according to the position which the hemorrhoid occupies. In the purely external variety, it is perfectly safe to cut them off, for there is usually no hem orrhage, or, if there is, it can easily be controlled. In the purely internal variety, it is safer to tie without cutting, and push the mass above the sphincter. In the mixed pile it is best to tie, but the portion external to the ligature may be cut away. In this form, before tying the ligature, a groove should be cut in the skin for the reception of the ligature; for these piles have their nerve supply near the skin and their vascular supply near the mucous membrane. By cutting through the skin, the amount of pain is diminished, and by not cutting the mucous membrane the risk of hemorrhage is avoided. It is usually necessary to surround the anus by a series of ligatures so as to include all of the hemorrhoids, for if any are allowed to remain they will probably develop, and the operation will have to be repeated.

The after treatment consists in the application to the anus of a cloth constantly wet with cold water; immediately after the operation, an opium suppository will be introduced into the bowel. When the ligatures separate, at the end of a week or ten days, there will remain only small ulcers, the healing of which may be hastened by the occasional application of nitrate of silver. This operation is apt to be followed by retention of urine, but this is less likely to occur if the precaution of stretching the sphincter is taken. The catheter has often to be used for five or six days. When the ligatures come away, the retention disappears.

(The doctor then removed several piles of the mixed variety, transfixing them, tying in two sections and cutting away the projecting portion).

